# The intervention process in the European Fans in Training (EuroFIT) trial: a mixed method protocol for evaluation

**DOI:** 10.1186/s13063-017-2095-0

**Published:** 2017-07-27

**Authors:** I. van de Glind, C. Bunn, C. M. Gray, K. Hunt, E. Andersen, J. Jelsma, H. Morgan, H. Pereira, G. Roberts, J. Rooksby, Ø. Røynesdal, M. Silva, M. Sorensen, S. Treweek, T. van Achterberg, H. van der Ploeg, F. van Nassau, M. Nijhuis-van der Sanden, S. Wyke

**Affiliations:** 1 Radboud Institute for Health Sciences, Scientific Center for Quality of Healthcare (IQ healthcare), Radboud University Medical Center, P.O. Box 9101, 6500 HB Nijmegen, The Netherlands; 2 Institute of Health and Wellbeing, College of Social Sciences, 27 Bute Gardens, University of Glasgow, Glasgow, G12 8RS UK; 3 MRC/CSO Social and Public Health Sciences Unit, Institute of Health and Wellbeing, 200 Renfield St, University of Glasgow, Glasgow, G2 3QB UK; 4 Department of Coaching and Psychology, Norwegian School of Sport Sciences, Oslo, Norway; 5 Department of Public and Occupational Health, and EMGO Institute for Health and Care Research, VU University Medical Center, Van der Boechorststraat 7, Amsterdam, 1081 BT The Netherlands; 6 Health Services Research Unit, University of Aberdeen, Aberdeen, AB25 2ZD UK; 7 Interdisciplinary Center for the Study of Human Performance (CIPER), Faculty of Human Kinetics, University of Lisbon, Estrada da Costa, 1495-688 Cruz Quebrada, Portugal; 8 School of Computing Science, University of Glasgow, Glasgow, G12 8RZ UK; 9 KU Leuven, Department of Public Health and Primary Care, Academic Centre for Nursing and Midwifery, Leuven, Belgium

**Keywords:** Process evaluation, Complex intervention, Health promotion, Public health, Sedentary lifestyle, Physical activity, Diet, Behaviour change, Football, Men’s health, Masculinity, Sports stadia

## Abstract

**Background:**

EuroFIT is a gender-sensitised, health and lifestyle program targeting physical activity, sedentary time and dietary behaviours in men. The delivery of the program in football clubs, led by the clubs’ community coaches, is designed to both attract and engage men in lifestyle change through an interest in football or loyalty to the club they support. The EuroFIT program will be evaluated in a multicentre pragmatic randomised controlled trial (RCT), for which ~1000 overweight men, aged 30–65 years, will be recruited in 15 top professional football clubs in the Netherlands, Norway, Portugal and the UK. The process evaluation is designed to investigate how implementation within the RCT is achieved in the various football clubs and countries and the processes through which EuroFIT affects outcomes.

**Methods:**

This mixed methods evaluation is guided by the Medical Research Council (MRC) guidance for conducting process evaluations of complex interventions. Data will be collected in the intervention arm of the EuroFIT trial through: participant questionnaires (*n* = 500); attendance sheets and coach logs (*n* = 360); observations of sessions (*n* = 30); coach questionnaires (*n* = 30); usage logs from a novel device for self-monitoring physical activity and non-sedentary behaviour (SitFIT); an app-based game to promote social support for physical activity outside program sessions (MatchFIT); interviews with coaches (*n* = 15); football club representatives (*n* = 15); and focus groups with participants (*n* = 30). Written standard operating procedures are used to ensure quality and consistency in data collection and analysis across the participating countries. Data will be analysed thematically within datasets and overall synthesis of findings will address the processes through which the program is implemented in various countries and clubs and through which it affects outcomes, with careful attention to the context of the football club.

**Discussion:**

The process evaluation will provide a comprehensive account of what was necessary to implement the EuroFIT program in professional football clubs within a trial setting and how outcomes were affected by the program. This will allow us to re-appraise the program’s conceptual base, optimise the program for post-trial implementation and roll out, and offer suggestions for the development and implementation of future initiatives to promote health and wellbeing through professional sports clubs.

**Trial Registration:**

ISRCTN81935608. Registered on 16 June 2015.

## Background

Robust evidence on whether interventions to improve public health are effective is essential. However, as many authors have argued, it is equally important to understand why particular interventions succeed or fail, under which circumstances and whether their effects can be reproduced so that better programs can be developed and implemented more widely [1–5]. A process evaluation, embedded within a randomised controlled trial (RCT), can provide this insight. If an intervention is successful, a process evaluation can illuminate the processes through which the intervention affects outcomes; if an intervention is not successful, a process evaluation can shed light on whether there was a failure of implementation or of the intervention itself. Either way, the learning from trial delivery of an intervention is maximised [6, 7].

In addition, programs which have been shown to be effective in a trial can be hard to implement more widely or can fail to show effects outside of the specific context of the trial, in part because of the way in which they are developed or implemented [8–12], particularly in public health [13–15]. Often, too little attention is paid to the detailed requirements that programs demand of the settings in which they are to be applied [16–18]. A process evaluation can provide detailed examination of the barriers and facilitators that influence the delivery of the program within different organisational settings and cultural contexts. It can describe what is necessary or desirable to set up a program within organisations and maximise its uptake in the target population; this is essential for planning for wider dissemination and implementation of a program should it prove successful.

In this paper, we present the protocol for a process evaluation of the implementation of the EuroFIT health and lifestyle program embedded within a pragmatic RCT [19]. The overall aim of the process evaluation is to investigate: (1) how the implementation is achieved in the various football clubs and countries; and (2) the processes through which the EuroFIT program affects outcomes. Before outlining the process evaluation framework and the methods we will use, we briefly describe the EuroFIT program itself.

## The EuroFIT program

EuroFIT is an evidence-based and theory-based, gender-sensitised, health and lifestyle program targeting physical activity, sedentary time and dietary behaviours in men. EuroFIT is informed by the Football Fans in Training (FFIT) program, which was developed and delivered in Scotland [20, 21]. The key concept in both FFIT and EuroFIT is to attract men to lifestyle change through an interest in football and their personal connections and loyalties to the football club they support. A pragmatic RCT of FFIT, conducted in 2011–2012, demonstrated that the program reached men at high risk of ill-health (according to their BMI and waist circumference) [22] and was successful in helping men lose weight and maintain that loss to 12 months (primary outcome) and in supporting positive changes in additional secondary outcomes [23]. Analyses of qualitative data collected as part of the FFIT research have elucidated some important mechanisms underlying this success, including the context, content and style of delivery; the opportunity to interact with men ‘like them’; learning skills for behaviour change; the chance to renegotiate aspects of their masculinity and rehearse healthier practices; the role played by family members in behaviour change; and access to an important cultural institution (the football club) [21, 24–26].

The EuroFIT program shifts focus from weight management (FFIT) to increasing physical activity and reducing sedentary time. To support this change in emphasis, participants are provided with a SitFIT™ device and access to a game called MatchFIT, both of which have been developed as part of the EuroFIT project. The SitFIT™ is a self-monitoring device that allows real-time self-monitoring not only of physical activity (through step counts), but also of sedentary behaviour (sitting time) and non-sedentary behaviour (upright time). MatchFIT is an app-based game designed to encourage social support around physical activity between sessions and after the end of the program. Both EuroFIT and FFIT aim to support participants in their improving diet.

**Fig. 1:**
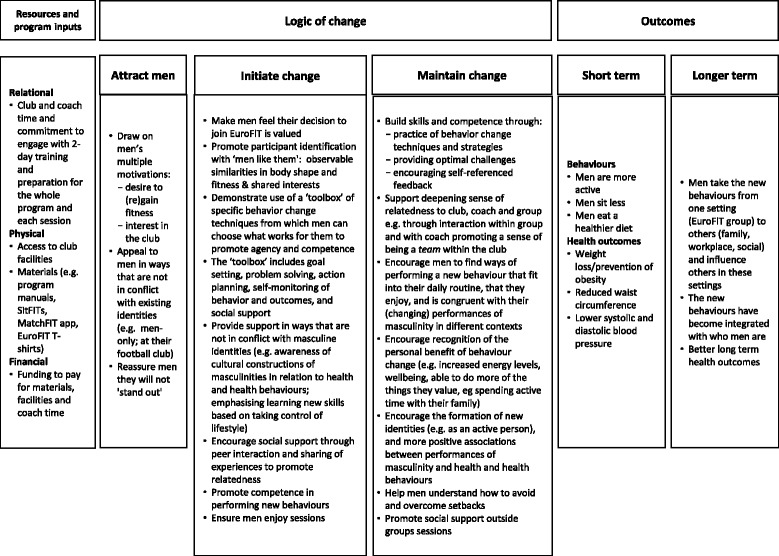
Logic model of the EuroFIT program

The effectiveness of EuroFIT will be evaluated in a multicentre pragmatic trial in 15 top professional football clubs across four European countries (the Netherlands, Norway, Portugal and the UK) [19]. For this trial, 1000 overweight men aged 30–65 years will be recruited and individually randomised within clubs. The primary outcome measures for this trial are changes in total physical activity (i.e. steps per day) and total sedentary time (i.e. minutes per day spent sitting), as measured by the activPAL device. Both outcomes are assessed over 12 months. The study is powered to detect an average increase of at least 1000 steps per day and an average decrease of at least 25 min per day spent sitting [19]. Cost effectiveness will also be investigated (Table 1, Fig. 1).

**Table 1:** The EuroFIT program details

Name (1)	EuroFIT
Why (2)	Lifestyle interventions targeting physical activity, sedentary time and dietary behaviours have the potential to support behavioural change and result in attainment or maintenance of healthier weight and other public health gains. Although men have often been reluctant to engage in such lifestyle programs, many are at high risk of long-term mental and physical health problems, as a result of high amount of sedentary time and lack of physical activity, poor diet and excess weight. The aim of the EuroFIT program is to help men, aged 30–65 years, with self-reported BMI of 27 or above to become more physically active and less sedentary, improve their diet, and maintain these changes over the long term. EuroFIT is designed to attract men by drawing on multiple motivations to do something: their desire to (re)gain fitness and/or an interest in football or their football club.
What, materials (3)	EuroFIT draws explicitly on motivational theories (Self-Determination Theory [41] and Achievement Goal Theory [42]) to encourage men to develop an internalised self-relevant motivation for becoming more physically active, sitting less and eating a healthier diet. It is also informed by sociological theories, including how practices of masculinity in different contexts and gendered identities intersect with health related-behaviours [25]. Access to club facilities for weekly group discussion sessions and physical activity training are required. Coach delivery manuals include the rationale, content and suggestions of how to deliver each weekly session. Participants receive: a manual including information and self-monitoring forms; a novel device, the SitFIT to allow self-monitoring of daily steps and non-sedentary behaviours (time spent upright/walking); and access to a new app-based game (MatchFIT) to promote social support and interaction around physical activity outside EuroFIT sessions.
What, procedure (4)	**Football clubs**: Football clubs decide to offer the EuroFIT program. **Training**: Club community coaches are trained over two days to deliver the program. Training is experiential and interactive and focuses on the ethos of the EuroFIT program; the reasons for becoming more physically active and less sedentary; the importance of ‘healthy standing’; promoting the use of self-monitoring, goal-setting, problem-solving and action-planning; supporting men’s motivation to sustain behaviour changes; facilitating group discussions; providing an environment which values men and their efforts, supports autonomy and encourages social support and a sense of relatedness; providing safe and optimally challenging physical activity. **Recruitment**: Clubs recruit participants through any of: online publicity (e.g. advertising on club/fan websites), e-mail, newsletter or social media announcements (i.e. Twitter, Facebook), poster/flyers, match-day advertising, face-to-face recruitment at home matches (handing out leaflets and collecting contact details), local/national media coverage, active involvement of local supporters’ organisations and word of mouth. **Content**: The EuroFIT program focuses first on initiating and then on maintaining behaviour change. In the early weeks, participants’ identification with ‘men like me’ is promoted (e.g. everyone receives a EuroFIT T-shirt, similar interests are emphasised). The coaches provide the men with support to change their behaviours that may challenge their masculine identities, but is not in conflict with them (e.g. emphasising learning new skills based on evidence of how to take control of lifestyle). Peer interaction, sharing of experiences and enjoyment is promoted during each session. Men are taught interactively how to use a ‘toolbox’ of specific behaviour change techniques and encouraged to try and use what works and is likely to be sustainable, for them. The ‘toolbox’ includes information on health and lifestyle presented in a way that is personally relevant and accessible, behavioural and outcome goal-setting, problem-solving, action-planning and self-monitoring of behaviour and outcomes (using a novel self-monitoring device, sitFIT, which provides feedback in real time on sedentary time and step counts), as well as building social support within (including using social media) and beyond the group (e.g. family, friends, work colleagues). The sessions also include physical activity training using club facilities, where coaches encourage each participant to work at an intensity that is appropriate for his own fitness and ability. At a later stage in the programme, men are helped to maintain changes by integrating the behaviour change strategies that they felt useful and significant for them, into their daily lives (goal-setting, self-monitoring, action-planning) and by using relapse prevention techniques. Coaches encourage vicarious learning of strategies for maintaining changes through interaction among group members and promote a deepening sense of relatedness to the club, the coach and the other men (e.g. becoming a team within the club, using the game-based app MatchFIT). Personal meaning of the changes already made is also prompted, indeed, men are encouraged to find ways of performing a new behaviour that fits into their daily routines and that they enjoy and to recognise the personally relevant benefits of behaviour change (e.g. increased energy levels, wellbeing, able to do more of the things which they themselves value, such as increased quality time with their family).
Who provides (5); How (6); Where (7); How much (8)	Professional football club community coaches deliver 12 weekly, face-to-face 90-min sessions to groups of 15–20 men. One reunion session is held 6–9 months after baseline. The sessions are held in club stadia and/or the clubs training facilities to foster an ‘insider’ view, increased physical and symbolic proximity to the club, and hence an enhanced sense of relatedness to the club.

Numbers in parentheses refer to the item number on the TIDieR checklist

European Fans in Training: TIDieR, Template for Intervention Description and Replication [27]

The EuroFIT program consists of 12 weekly, 90-min group sessions of 15–20 men delivered at professional football clubs by club community coaching staff. Each of the 15 EuroFIT clubs commit to deliver two EuroFIT groups. EuroFIT also includes one reunion session which is held at the club 6–9 months after the start of the program (the precise timing is determined by the coaches delivering the program). In the outcomes analysis, we will follow an intention-to-treat procedure [19]. For the purposes of this process evaluation, a participant will be considered to have ‘completed’ the EuroFIT program if they attend six or more of the 12 sessions (excluding the reunion session). Table 1 presents an overview of the program in relation to the international standard for describing interventions, the Template for Intervention Description and Replication (TIDieR) [27]. Figure 1 depicts the logic model of the program. The content of the logic model – the theory of how EuroFIT affects outcomes and how the program is implemented in the context of the football club – will be examined through 18 research objectives (ROs) as described in Table 2.

**Table 2:** Research objectives and data collection methods for the EuroFIT process evaluation

Research objective (RO)	Quantitative methods					Qualitative methods		
To understand:	Baseline, post program and 12-month questionnaires EuroFIT participants (n = 500)	Structured telephone questionnaire participants opting out of the study and/or program	Participant attendance sheets for each session (n = 360) and coach logbooks from each session (n = 360)	Coach questionnaires: post training and post program (n = 30)	Participants’ SitFIT and MatchFIT usage logs	Observation of sessions (n = 30)	Interviews with club representatives (n = 15) and coaches (n = 15)	Post-program and 12-month focus group discussions with EuroFIT participants (n = 30)
DOMAIN 1: IMPLEMENTATION OF THE EUROFIT PROGRAM								
How the delivery is achieved (ROs 1–4)								
1. Sources and procedures for recruitment of clubs and reported decision-making in clubs in relation to participating in EuroFIT.	X						X	
2. Sources and procedures for recruitment of coaches to deliver the EuroFIT program in participating clubs.							X	
3. Experiences of coach training and its usefulness in program delivery.				X			X	
4. Sources and procedures for recruitment of participants.	X						X	X
What is delivered (ROs 5–7)								
5. Participation in the EuroFIT program including number of sessions attended and the reported extent to which SitFIT and MatchFIT were used.	X	X	X	X	X			X
6. The number of sessions and key elements of the EuroFIT program that were delivered by coaches.			X					
7. The extent to which coaches delivered the EuroFIT program according to the coach manual and training.			X	X		X		
Reach (RO8)								
8. The characteristics of participants who were attracted to and recruited to EuroFIT in relation to demographic and health risk profile.	X							
DOMAIN 2: MECHANISMS OF IMPACT (ROs 9–15)								
9. Participants’ reported reasons for joining, continuing with or opting out of the EuroFIT program.	X	X						X
10. Interaction between men and between men and coaches during the program.						X		
11. How coaches used the coach manual and associated materials to deliver the EuroFIT program.						X	X	
12. Coaches’ views and experiences of the EuroFIT program and materials, particularly which elements of the program were viewed as helpful and unhelpful in supporting participants to make lifestyle changes.							X	
13. Participants’ views and experiences of the EuroFIT program and materials, which elements of the program were viewed as helpful and unhelpful in supporting them to make changes and how the environment coaches created influenced participant responses. We will pay particular attention to the use of the toolbox of behaviour-change techniques including SitFIT, MatchFIT, goal-setting and self-monitoring.	X							X
14. Participants’ experiences of maintaining (or not) any lifestyle changes made as a result of the program 12 months after baseline.								X
15. Participants’ views of which aspects of the program that were helpful and which less so for supporting long-term change.	X	X						X
DOMAIN 3: CONTEXT OF THE PROFESSIONAL FOOTBALL CLUB (ROs 16–18)								
16. The characteristics of football clubs that decided to participate in EuroFIT in relation to league, annual income, fan base, past experience of delivering health promotion programs and facilities available in the club.							X	
17. The characteristics of coaches that delivered EuroFIT in relation to background, demographic characteristics, skills and experiences.				X			X	
18. The perceived barriers and facilitators to implementing the program in the clubs, including what attracted club management to taking part in the delivery of EuroFIT, what worked well and less well in terms of recruitment of participants, recruitment and training of staff, organisation of the sessions and delivery of the program week on week, and future activities and intention to use the EuroFIT program			X	X			X	

### Conceptual framework for the process evaluation

**Fig. 2:**
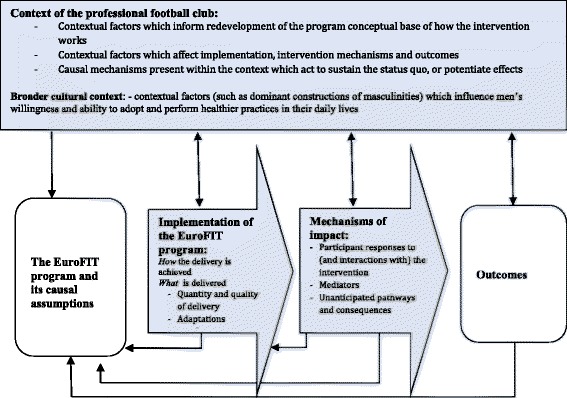
Key functions of EuroFIT process evaluation (in *blue*). Adapted From: Moore et al. 2015 [6]

The EuroFIT program has multiple interacting components [1, 6, 28]. The design of this process evaluation has been informed by recent Medical Research Council (MRC) guidance on the process evaluation of complex interventions [1, 6] which identifies three essential features of understanding the process through which outcomes are achieved: context, implementation and mechanisms of impact (the rectangular and arrow-shaped boxes in Fig. 2).

For the purposes of the process evaluation we describe context, implementation and mechanisms of impact as follows [1]:

- **Context**: An examination of how the broader cultural context of the country and the specific cultural context of the football club into which EuroFIT is introduced, influences and interacts with the delivery and functioning of the program components.
- **Implementation of the EuroFIT program**: An examination of how EuroFIT delivery is achieved and what is actually delivered. We will describe the structures, resources and processes through which EuroFIT delivery is achieved, the extent to which EuroFIT was delivered as intended, any adaptations made to the program and the sociodemographic characteristics of the participants recruited to the program.
- **Mechanisms of impact**: An examination of the processes through which the program affects outcomes through understanding how participants and coaches respond to and interact with EuroFIT and how the intervention supports change (or not).

We have structured the 18 ROs for this process evaluation around these three domains: Table 2 details eight ROs related to the implementation domain ROs 1–8), seven in relation to the mechanisms domain (ROs 9–15) and three in relation to the context domain (ROs 16–18). Achievement of these objectives will allow us both to interrogate and evaluate the logic model and to assess the intervention using a standardised process evaluation framework [1]. It will also enable us to address the twin aims of the process evaluation, which are to investigate: (1) how the implementation is achieved in football clubs and countries; and (2) the processes through which the EuroFIT program affects outcomes.

## Methods

### Overall study design

This mixed methods process evaluation is embedded in the main EuroFIT trial, which is being conducted in the Netherlands, Norway, Portugal and the UK [19]. The process evaluation will be conducted by researchers from University of Glasgow, University of Aberdeen, KU Leuven, University of Lisbon, Norwegian School of Sport Sciences, Radboud University Medical Centre Nijmegen and VU University Medical Centre. The process evaluation is coordinated by the University of Glasgow in the UK (further referred to as the process evaluation lead). Because this process evaluation is embedded in the trial, a process evaluation subgroup has been formed consisting of researchers from each country, thereby making explicit who is involved in the collection and analysis of process data. This subgroup will be further referred to as the process evaluation team. Written standard operating procedures have been created for all of the data collection activities described in this paper. Members of the process evaluation team will receive training in data collection and analysis procedures over two days to harmonise approaches across countries.

Ethical approval for the process evaluation has been obtained from the appropriate country-specific ethics committees (Ethics committee of the VU University Medical Center [2015.184]; regional committees for medical and health research ethics, Norway [2015/1862]; Ethics Council of the Faculty of Human Kinetics, University of Lisbon [CEFMH 36/2015]; and Ethics Committee at the University of Glasgow College of Medicine, Veterinary and Life Sciences [UK] [200140174]).

### Quantitative data collection

Quantitative data will be collected through participant questionnaires in the intervention arm of the trial (n = 500), coach questionnaires (n = 30), attendance sheets and coach logs of sessions delivered (n = 360) and participants’ logs from the SitFIT and the game-based app MatchFIT. To minimise the respondent burden, questions relevant to the process evaluation will be added to participant questionnaires (for intervention group members only) which collect trial outcome data. The Robertson Centre for Biostatistics (RCB) within the University of Glasgow provides data management in support of delivering the trial [19]. All trial subject data will be entered via a study web portal (an online data entry system) with data validation checks. Where applicable, data will be collected in the local language and entered into the study web portal in the local language. Standard operating procedures (SOPs) developed as part of the trial [19] are used to guide quantitative data collection for the process evaluation. Each of the quantitative methods is explained below including which ROs they are relevant to (Table 2). We will refer to the objectives using numbers (ROs 1–18).

#### Participant questionnaires

The baseline participant questionnaire will gather information on the characteristics of the participants who were recruited to EuroFIT (demographics and health risk profile), how they heard about the program and why they joined (ROs 4, 8 and 9). The post-program participant questionnaire (intervention group only) will contain questions about attendance at sessions, experiences of the program, perceived competence of the coaches delivering the program and the environment they created, and the extent to which participants used the activities and tools from EuroFIT while taking part in the program (ROs 5 and 13). The 12-month participant questionnaire will contain questions for the intervention group on the extent to which participants remain in contact with the other men and the coaches who took part in the EuroFIT program at their club and on the extent to which (behavioural and technological) tools from the EuroFIT program are still being used after the program has ended (RO 15).

#### Telephone questionnaire with participants opting out

If participants withdraw from the trial (measurements) or drop out of the program, their reason for leaving will be assessed via structured telephone interviews (RO 9).

#### Usage data from SitFIT and MatchFIT

Data are collected remotely from users of the SitFIT and MatchFIT and will include logs of data uploads, error reports, user clicks, logins and logouts (RO 5). Each item of logged data includes a timestamp, information about the web browser and device type being used, and (except for pre-registration and pre-login activities in MatchFIT) a unique SitFIT identifier. As part of the post-processing of the data, the SitFIT identifier will be linked to the participants’ ID, usernames will be replaced with a pseudonym and the text of the personal annotations in MatchFIT will be replaced with an integer representing how much text was written.

#### Attendance sheets and log of sessions delivered

Club coaches will be asked to keep an online record of attendance at each of the 12 weekly sessions and the reunion session, to be completed as soon after each session as practicable (RO 5). The program will be delivered to two groups of approximately 15–20 men in each of the 15 participating football clubs. During the course of the trial, a total of 360 EuroFIT sessions will take place. In addition, coaches will be asked to complete an online log to assess their overall view of each session, to record the time they spent on preparation and delivery of the session, and to record the extent to which they consider that intervention components were delivered as intended or not (self-report). In addition, coaches will be asked to provide feedback on the session through a set of open ended questions in terms of what worked well and what did not work well (ROs 6, 7 and 18).

#### Coach questionnaires

An evaluation questionnaire will be administered to coaches after the two-day intervention training (RO 3). After the last program session has been delivered, all coaches will be asked to complete an online questionnaire (ROs 3, 7, 12 and 17). This questionnaire contains an overall evaluation of the program and questions asking how often coaches used the activities and tools that are specific to the program.

### Qualitative data collection

Observations of sessions, interviews and focus group discussions will be conducted. SOPs have been written for the qualitative data collection and analysis, based on templates provided by the ACT consortium [29, 30] and the HAND-OVER consortium [31]. The process evaluation team are trained according to these procedures. Regular team debriefing meetings will be organised, during which the process evaluation team will: discuss the qualitative data collection and analysis, and any problems or suggested changes to topic guides (e.g. to pursue emerging themes); and consider any difficulties with the procedures, and possible actions to ameliorate these difficulties. In the team debriefings, the process evaluation lead will keep track of the progress of the data collection and analysis in the four countries.

#### Interviews with club coaches and club representatives

Semi-structured interviews will be conducted with club community managers and club community coaches at the participating football clubs (ROs 1–4, 11, 12, 16–18). The purpose of the interviews is to find out what coaches and club representatives think of the program, their experiences of what worked or did not work with regard to getting the program up and running, barriers and facilitators for implementation, recruiting men for the program, delivering the program from week to week, whether or not they see a future for the program in the club and why, and what would be needed in the future to ensure that they continue with the program. The interviews will explicitly aim to discuss barriers and facilitators for implementation as well as capture contextual issues (such as availability of facilities) that may have shaped the delivery of the program. Club community managers and coaches will be eligible to participate in an interview if they have been personally involved in the recruitment, delivery or organisation of sessions of the EuroFIT program at their club. We plan to complete 15 interviews with club community managers and 15 interviews with coaches who delivered the program in the participating football clubs (30 interviews in total). This sampling strategy enables us to obtain views and actual experiences from each participating club across the four countries. Interviews will be conducted by a trained member of the process evaluation team not directly involved in the training of the coaches and who is based in the same country as the football club. A topic guide has been developed, will be piloted in the first few interviews and will be adapted, through discussion with the process evaluation subgroup, where necessary. Interviews will be audio-recorded with permission from the interviewee. The researcher will also take brief notes during the interviews.

#### Observations of EuroFIT sessions

Researchers will observe a sample of deliveries of the EuroFIT program at each participating club to obtain data on how men interact with one another, how they respond to different elements of the program, how they interact with the coaches that lead the sessions and any features of the interaction that might influence program effects (ROs 7, 10 and 11). We have chosen not to observe sessions 1 and 2, in order to allow groups to ‘form’ without adding the anxiety of being observed by researchers [32]. We will observe the delivery of two sessions in each of the participating football clubs (30 sessions in total). Session 4 will be observed in every club, because it covers multiple topics and activities that relate to the main targets of the program (physical activity, sedentary lifestyles, self-monitoring, goal-setting and action-planning). Program sessions 3 and 5–12 will be observed at least once in one of the 15 clubs. This sampling strategy will allow us to observe the delivery of each session at least once (with the exception of sessions 1 and 2) and to make comparisons of delivery style and interactions for the same session across the clubs and countries. A trained member of the process evaluation team who is based in the same country as the football club being observed will conduct the observations. Observations will be conducted in a non-participative manner, as far as is possible, to limit interference with the intervention delivery. After the observation is complete, the observer will use all notes taken during the observation to write a ‘thick description’ of the session (a detailed account of the session that is being observed and the experiences in the field, that attempts to convey the ‘meaning’ of an observation, going beyond simple factual accounts only) [33, 34].

#### Focus groups

We will conduct focus groups with EuroFIT participants at each football club. The purpose of the focus groups is to find out what men think of the EuroFIT program, any impacts it has had on their lives, which elements of the program they viewed as helpful and unhelpful in supporting them to make changes and any suggested changes to the program (ROs 4, 9, 13–15). The first focus groups will take place after the 12-week program has ended and after the post-program measurements for the trial have been completed. In these post-program focus groups, we will be particularly interested in participants’ reasons for joining and continuing with the EuroFIT program; their views and experiences of the program and materials; any impacts they feel that taking part has had on their lives (including changes they have made to their behaviours); and which elements of the program were viewed as helpful and unhelpful in supporting them to make changes.

The second set of focus groups will be conducted after the 12-month trial measurements have been completed and aim to understand men’s experiences after 12 months of attempting to make and maintain (or not) any changes made as a result of participating in the EuroFIT program; what aspects of the program were helpful/less helpful for supporting long-term change; what barriers and facilitators they have experienced to making/maintaining change; the extent to which the changes have become routinised in their daily lives and integrated with their (reformulated) identities; and the responses of their family, social and work colleagues to their changed behaviours, appearance and wellbeing.

The session attendance sheets compiled by the EuroFIT coaches will be used to sample participants for the post-programme focus groups. To be invited for a focus group, men must have attended at least half of the 12 program sessions, because we think it is important that the focus group participants can share their views based on substantial experiences of the program. For each focus group, we aim to recruit 6–8 participants, drawn from both delivery groups, who are of varied ages.

Each focus group will take place at the football club and will be conducted by a moderator, who will be fully trained to ensure consistency across countries. Where possible, the moderator will be assisted by a note-taker (reporting the setting and atmosphere during the focus groups and any significant interactions or non-verbal exchanges between participants). An audio-recording will be made, with participants’ permission. Men will receive a €20/£20 voucher in return for their participation.

### Data collection pilot

Elements of this process evaluation were tested in one football club in the UK which offered a pilot version of the EuroFIT program in 2015. We conducted observations of this delivery of the 12 EuroFIT program sessions and used a standardised observation proforma. We also piloted the format of the coach log data collection instrument. The participants and coaches who took part all found the methods acceptable. Based on this study, we made small adjustments to the coach logs and the observation proforma.

### Quantitative data analysis

Descriptive statistics (mean, SD, proportions) will be used to report participants’ and coaches’ characteristics and results of pre-structured questions from the questionnaires. Data from the attendance sheets and coach logs will be summarised using this method. Descriptive summaries and charts will be produced to present attendance, sessions delivered and key components delivered, to help identify patterns of incomplete delivery. In addition, estimates of the total program hours received by participants will be computed per club using data from the attendance sheets. All reported (suggestions and reasons for) adaptations to the program and any other answers to open ended questions will be listed, analysed and summarised. The results will be translated into English by a local member of the process evaluation team and reported back in English to the process evaluation lead. Descriptive statistics and visualisations will also be used to explore and report the use of the SitFIT and MatchFIT. The key performance metrics are uptake and retention rate (measured in terms of weekly active users). The use of individual features of MatchFIT will also be explored.

### Qualitative data analysis

All note-taking during observations, and all interviews and focus groups, will be conducted in the local language; all data will be transcribed in the local language of the club delivering the program [31]. Transcription of interview and focus group data will be done verbatim (every word captured exactly). A standard layout will be applied to all transcripts. After checking for accuracy, each final anonymised transcript will be added to the analysis.

A structured thematic, framework approach will be used to analyse the qualitative data. The analysis will consist of five steps [35], with each country initially working independently, and will be framed by the ROs.

#### Step 1: local familiarisation and initial coding

Within each country, three files (i.e. focus group transcripts, interview transcripts or observation thick descriptions) will be read line by line by a member of the process evaluation team with expertise in qualitative research. For each line or subsection that carries meaning in relation to the ROs or unanticipated issues, the text will be highlighted and assigned a code. All the codes will be described in English. In each country, a second independent coder will read and check the codes. Any disagreement will be resolved through discussion; if necessary, a third local researcher will be consulted to aid resolution of any disagreement in the interpretation of the coding.

#### Step 2: developing a consolidated codebook

A face-to-face meeting of the process evaluation team will be organised, during which the initial local codes, derived from the three selected files, will be shared and discussed. A researcher from the consortium who is expert in qualitative methods, and not directly involved in data collection and coding, will chair this meeting. During this meeting, we will create an affinity diagram where we will separately record each suggested code on a post-it note, assemble similar suggested codes into groups, discard duplicate codes and assign groups of similar codes an adequate descriptive label [31]. During this meeting, a consolidated codebook (coding template) will be developed for each relevant RO.

#### Step 3: coding and indexing

The coding of the first three files will then be adjusted according to the consolidated codebook. The remaining files will also be coded against the consolidated codebook. Regular conference calls, email and, if necessary, face-to-face meetings will be organised to adjust and refine the codebook. When all the data have been coded, each country will provide the process evaluation lead with a report in English that summarises the key findings for each RO, following a standard template.

#### Step 4: charting

In dialogue with the process evaluation team, the process evaluation lead will analyse the country specific reports and write overall findings paying attention to any cultural and other differences in sub-groups and countries.

#### Step 5: synthesis and drawing conclusions

A draft set of findings and conclusions will be circulated to the process evaluation team and the consortium for feedback.

### Mixed methods analysis

Once qualitative and quantitate analyses are complete, we will compare and integrate findings from different data sources to answer the 18 ROs. The integration will be guided by the ‘triangulation protocol’, which suggests researchers assess degrees of agreement and dissonance across the datasets and also to identify areas of ‘silence’, i.e. where a given dataset has nothing to contribute [36, 37]. A convergence coding matrix, summarising findings for each country will be constructed, based on Table 2, ensuring that all relevant data sources contribute to assigned ROs. These four matrices (one for each of the four countries) will form the focal point of the six steps of the triangulation protocol method. We will then use the country-specific matrices to produce a convergence matrix that covers all four EuroFIT sites.

Following Moore et al.’s model, the finalised convergence matrix will be used to establish the influence of context, implementation and mechanisms of action on the outcomes of EuroFIT. The finalised convergence matrix will also be used to assess the extent to which the logic model, and the causal assumptions it contains, can explain the outcomes the program produced. The analysis will guide us to the key mechanisms that are essential for program success and the parts of the program that are difficult to implement (or even implementation failures). The synthesis of findings from the different data sources is an important step in interpreting program outcomes, understanding the context in which the program is delivered and identifying the mechanisms of impact or implementation difficulties.

## Discussion

This paper describes the design of a mixed methods process evaluation embedded within a multicentre pragmatic trial, for which 1000 overweight men, aged 30–65 years, will be recruited in 15 top professional football clubs across the Netherlands, Norway, Portugal and the UK. EuroFIT is an evidence-based and theory-based, gender-sensitised, health and lifestyle program that aims to attract men who wish to make changes to their physical activity, sedentary behaviour and diet, through an interest in football and their personal connections and loyalties to the football club they attend. The process evaluation is designed to investigate how the implementation is achieved in the various football clubs and countries and the processes through which the EuroFIT program affects outcomes. By publishing the protocol for this process evaluation, we make the methodological choices that we have made explicit.

Professional sporting venues, and in particular professional football clubs, are increasingly seen as promising sites for the delivery of health promotion programs because of their potential to reach people who might not otherwise consider taking part in a lifestyle program [21, 23, 38, 39]. Therefore, a thorough evaluation of the processes through which such programs are implemented and how they affect outcomes is important [40]. As this approach to public health improvement begins to spread globally, a significant strength of this process evaluation is that the four distinct national sites allow us to explore the difference that national, club and cultural contexts can make to the implementation, mechanisms of action and, ultimately, outcomes of the EuroFIT program.

While we have tried to be comprehensive in addressing all issues utilising different methods for data collection, we have had to make some compromises due to time and resource constraints. The number of observations of deliveries had to be kept to a maximum of two per club (30 sessions out of a possible 360). Observing more deliveries might enable a more nuanced understanding of how each component of the program is delivered and following one delivery group over the full 12-week program could also offer more insight into the process of group formation and group dynamics. The ongoing contact between researchers and the delivery of the intervention, through observations conducted during the trial, will allow researchers to be responsive and to construct topic guides for coach and club interviews and participant focus groups that reflect areas of interest that emerge. As described above, the ongoing contact will be mostly passive, as observers will work in a non-participative manner to limit interference with the program delivery during the trial.

Apart from the 30/360 sessions we are able to observe, we rely on the coaches to self-assess the ‘fidelity’ with which they deliver their weekly sessions. Although self-report presents some pragmatic advantages in terms of simplicity, convenience and affordability, we are well aware of the risk of social desirability bias. For instance, coaches may be reluctant to say that they did not deliver certain intervention components or they might not have the skills to rate their own competence [1]. The testing of these two methods of data collection– observations and coach logs – in a small-scale pilot delivery of the EuroFIT program, enabled us to incorporate some improvements in both procedures.

Despite these limitations, a balanced design for the process evaluation of EuroFIT in the context of a RCT has been achieved by following the MRC guidance on the conduct of process evaluations [1]. We therefore anticipate that we will be able to provide a comprehensive account of how outcomes were affected by the program, what is necessary to implement such a program in professional football clubs in several clubs and countries, and to evaluate the impact of contextual differences between sites. This will allow us to re-appraise the program’s conceptual base, contribute to any necessary improvements to the EuroFIT program and offer suggestions for the development, evaluation and implementation of future football-based and other professional sports-based initiatives to promote health and wellbeing.

## Abbreviations

ACT Consortium: artemisinin-based combination therapy Consortium
EuroFIT: European Fans in Training
FFIT: Football Fans in Training
MRC: Medical Research Council
RCT: Randomised controlled trial
RO: Research objective
SD: Standard deviation
TIDieR: Template for Intervention Description and Replication
